# Association between colorectal cancer testing and insurance type: Evidence from the Swiss Health Interview Survey 2012

**DOI:** 10.1016/j.pmedr.2020.101111

**Published:** 2020-05-04

**Authors:** Alexander Leonhard Braun, Anja Kässner, Lamprini Syrogiannouli, Kevin Selby, Jean-Luc Bulliard, Yonas Martin, Idris Guessous, Kali Tal, Cinzia Del Giovane, Marcel Zwahlen, Reto Auer

**Affiliations:** a Institute of Primary Health Care (BIHAM), University of Bern, Switzerland; b Institute of Social and Preventive Medicine (ISPM), University of Bern, Switzerland; c Center for Primary Care and Public Health (Unisanté), University of Lausanne, Switzerland; d Department of General Internal Medicine, Inselspital, Bern University Hospital, University of Bern, Switzerland; e Unit of Population Epidemiology, Department of Community Medicine and Primary Care and Emergency Medicine (UEP), Geneva University Hospitals, Switzerland

**Keywords:** Colorectal cancer screening, Switzerland, FOBT, Colonoscopy, Screening rates, Health insurance

## Abstract

Both colonoscopy and fecal occult blood test (FOBT) are commonly used for colorectal cancer (CRC) screening, but colonoscopy costs much more than FOBT. Swiss insurance offers high or low deductibles and choice of basic or private insurance. We hypothesized that high deductibles and basic insurance discourage colonoscopy, but do not change FOBT rates.

We determined the proportion of patients tested for CRC in Switzerland (colonoscopy within 10 years, FOBT within 2 years), and determined associations with health insurance type.

We extracted data on 50–75-year-olds from the Swiss Health Interview Surveys of 2012 to determine colonoscopy and FOBT testing rates (n = 7335). Multivariate logistic regression models estimated prevalence ratios (PRs) of CRC testing associated with health insurance type (deductible and private insurance), adjusted for socio-demographic factors (age, gender, education, income) and self-rated health.

The weighted proportion of individuals tested for CRC within recommended intervals was 39.5%. Testing with colonoscopy was significantly associated with private insurance (PR 1.85, 95% CI: 1.46–2.35) and low deductible (PR 2.00, 95% CI: 1.56–2.57). Testing with FOBT was significantly associated with deductible (PR 1.71, 95%CI:1.09–2.68) but not with private insurance.

About 60% of the Swiss population was not current with CRC testing. After adjusting for covariates, private insurance and low deductible was significantly associated with higher prevalence of CRC testing, indicating that waiving the deductible could increase CRC screening uptake and reduce health inequality.

## Highlights

• Colonoscopy and FOBT are both recommended for colorectal cancer screening.
• Colonoscopy costs much more, so test choice might be linked to insurance type.
• Private insurance and low deductibles were associated with more colonoscopies.
• FOBT, which is cheap, was not associated with private insurance.

## 1 Introduction

Colorectal cancer (CRC) is the third leading cause of cancer mortality in Switzerland, killing 1600 people annually (Arndt et al., 2016). Most of these lives could be saved by CRC screening (Brenner et al., 2014, Meester et al., 2015). The US Preventive Task Force (Bibbins-Domingo et al., 2016) and the European panel (Arditi et al., 2009) recommend screening for patients 50–75 years old with either colonoscopy every ten years or faecal occult blood test (FOBT) every 1–2 years. There are few studies investigating CRC screening in Switzerland, but existing studies find screening has been underused. Of patients who visited Swiss university primary care practices in 2005 and 2006, only 33.6% were tested at recommended intervals (Fischer et al., 2013). CRC testing rates are higher in the US, where, by 2015, 63% of the population had been tested (Schroy et al., 1997). A later study based on the Swiss Health Interview Survey (SHIS) reported screening rates among 50–75-year-olds were 18.9% in 2007 and 22.2% in 2012 (Fedewa et al., 2015). However, this study solely included tests performed for screening reasons. Diagnostic CRC testing may have a preventive effect, so both diagnostic testing and screening should be included in attempts to identify the eligible population never tested for CRC (Stock et al., 2011). In Switzerland, insurance only covered diagnostic tests for symptomatic patients before 2013, and not screening. In this period Switzerland lacked CRC screening programs that covered the whole cost so, before 2013, physicians or respondents may have had incentive to misreport the reason for CRC testing. Testing the impact of the change in this policy will not be possible until 2019, when the 2017 SIHS survey results become available. The goal of this study is to establish the baseline relationship between screening and insurance coverage before the 2013 change.

CRC testing rates have been associated with insurance type, a modifiable factor in use of effective preventive care (Hsia et al., 2000, Matthews et al., 2005, Weiss et al., 2013). High deductibles may be associated with lower CRC testing rates, especially for colonoscopy. The US Affordable Care Act (ACA) eliminated cost sharing for preventive screening in 2010 and though some studies found that eliminating costs did not change overall screening rates for colonoscopy (Mehta et al., 2015), others found that no-charge screening raised colonoscopy rates more among those with high-deductible health plans (HDHPs) (≥$1000) than among those with low-deductible health plans (≤$500) (Wharam et al., 2016). This trajectory may also be visible in Switzerland, where deductibles may impose financial barriers that reduce testing rates, especially for colonoscopy, which is much more expensive than FOBT. Insurance type is less likely to affect FOBT rates. The population in Switzerland with cheaper insurance plans and high deductibles might be lower in their colonoscopy rate.

Some patients in Switzerland can opt for semi-private or private insurance to cover hospital services beyond those mandated by statutory health insurance such as free choice of hospital doctor or better hospital accommodations (Biller-Andorno and Zeltner, 2015). Before 2013, some supplementary insurance packages covered CRC screening, but researchers have not tested the association between type of insurance and CRC testing or adjusted for other factors associated with type of insurance, like healthcare utilisation and income.

We thus determined the proportion of respondents tested for CRC in Switzerland with colonoscopy within ten years or FOBT within two years, and identified associations between testing and health insurance type by reanalysing data from the SHIS from 2012. We explored differential associations between self-reported reasons for CRC testing, including screening and diagnostic CRC, and insurance type.

## 2 Methods

### 2.1 Data source

Since 1992, and every five years thereafter, the Swiss Federal Statistics Office (SFSO) has conducted the cross-sectional, nationwide, population-based Swiss Health Interview Survey (SHIS). The SHIS sample represents the Swiss resident German-, French-, and Italian-speaking population, aged 15 years and older, who live in private households. The survey comprises a telephone interview (Part One) and an online or hard copy postal survey (Part Two). SFSO invites all participants to take both surveys; we included only those who had completed both.

The 2012 survey used the SFSO’s population register-based individual sampling frame (Swiss Federal Statistics Office (SFSO), 2013), and stratified random sampling by canton and weighted each observation by region, household size, age, sex, and nationality. SFSO’s weights assured data represented the Swiss resident population. The SFSO collected, anonymized and shared data according to the Swiss Federal Statistics Act (The Federal Council, 1993), so our study did not require ethical approval given that it fell outside of the scope of the Swiss Human Research Act.

### 2.2 Study variables

Our outcome of interest was type of CRC test: an FOBT only within the last two years or a colonoscopy (with or without FOBT) within the last ten years. We derived our outcomes from two survey questions (originally in German, Italian and French): 1) “Have you ever had a faecal occult blood test (FOBT)?” 2) “Have you ever had a lower gastrointestinal endoscopy?” Each question was followed up with 3) “What was the reason for your last examination?” (screening, diagnostics, clinical follow-up) and, 4) “What was the date of your last examination?” If the respondent could not supply the month and year, they were asked, 5) “Were you examined within the last year?” The survey did not ask respondents to distinguish between FOBTs (haemoccult [guaiac-based] and faecal immunochemical [FIT]) tests.

To derive our co-variates, we extracted SHIS survey data on socioeconomic and health type, health-related behaviour, and healthcare use. We chose the covariates of interest based on 1) face validity, 2) a review of the literature on determinants of CRC testing and 3) a predefined directed acyclic graph (DAG) (Supplementary File 1). We extracted information on type of insurance and deductible from answers to 1) “What is your health insurance coverage for a hospital stay?” (basic, semi-private, and private) and 2) “How high is your annual deductible?” (300 CHF; 500, 1000 or 1500 CHF; 2000 or 2500 CHF). To assess self-rating of health we extracted answers to “What is your general health status?” (very good; good; moderate; bad; very bad). We determined household income from the question, “How high is your total monthly net income, minus social security taxes and pension contributions,” based on SFSO quartiles (<2521; 2521–3599; 3600–5199; >5200 CHF). We used the SFSO assessment of education to determine level of education, grouped into three categories (primary, secondary, tertiary) that correspond to the international standard classification of education (UNESCO, 2012). We also considered sex, age (50–59; 60–69; 70–75), and nationality (Swiss or foreign).

### 2.3 Statistical analysis

Descriptive statistics indicated proportions of the population tested by FOBT within the last two years, colonoscopy within the last ten years, and combination of tests. Our analysis accounted for SFSO weights. We used the Wald test to calculate p-values for differences in testing methods between the two survey years. We described characteristics of the study population, categorized by year and covariates. We calculated percentages to describe the characteristics of responders and the proportions of people tested with FOBT only or colonoscopy (with and without FOBT) by survey year. We used the Chi-square test to evaluate the association between categorical variables. In each survey year, we used the multivariate multinomial logistic regression model to analyse the association between CRC testing and health insurance type. Prior to testing our study hypotheses on the association between non-modifiable factors and modifiable factors on CRC testing in the dataset, we draw a directed acyclic graph on the hypothesized associations (see Supplemental File 1). We adjusted models for all covariates. We estimated the prevalence ratio (PR) of each type of CRC testing and, with 95% confidence interval (CI), and compared multivariate multinomial logistic regression models. “No testing” was the baseline category in our model. We computed the marginal proportion of having been tested with either FOBT only or colonoscopy (with and without FOBT) at each level of type of insurance and deductible. We further tested if self-reported “screening” or “diagnostic” CRC tests altered the measures of association between CRC testing and the covariates in analyses stratified by type of test (colonoscopy vs. FOBT). Since participants may have changed deductible between the time they were tested for CRC and the time they were surveyed, we tested the sensitivity of our results to self-reported changes in insurance status. The SHIS did contain data on participants who reported they had changed deductible within the last 12 months, and we excluded them from the main analyses to see if that changed our outcome. Second, we restricted the outcome to colonoscopies performed within the last 12 months (Hamman and Kapinos, 2016). The threshold for statistical significance for all analyses was p < 0.05. We performed all analyses with Stata version 14 (Stata Corp, College Station, Texas, US).

## 3 Results

Of the 41,008 people selected to participate in the SHIS 2012, 18,357 completed both parts of the survey. We analysed data from 7342 50–75-year old participants. From these, we excluded 7 respondents for missing data on FOBT or colonoscopy (N = 7335). Table 1 shows weighted characteristics of respondents’ co-variates. Most respondents had the lowest deductible (48.3%) and few respondents had the highest deductible (2000–2500 CHF; 14.2%). Most participants had basic insurance (65.5%) rather than private insurance (10.0%).

**Table 1:** Characteristics of 50–75-year old respondents by survey year, from the Swiss Health Interview Survey 2012 (N = 7335).

	N = 7335
	%
Age (years)	
50–59	45.5
60–69	38.3
70–75	16.2
Sex	
Male	49.2
Female	50.8
Nationality	
Swiss	84.2
Not Swiss	15.8
Education	
Primary	16.4
Secondary	54.3
Tertiary	29.3
Income^1^	
<2521 CHF^2^	15.5
2521–3599 CHF	22.4
3600–5199 CHF	31.4
>5200 CHF	30.7
Self-rated health	
Very good	30.3
Good	46.3
Moderate	18.4
Bad	4.1
Very bad	0.9
Deductible	
300 CHF	48.3
500/1000/1500 CHF	37.5
2000/2500 CHF	14.2
Insurance	
Basic	65.5
Semi-private	24.4
Private	10.0

Note: Missing values: education = 24, income = 972, self-rated health status = 10, deductible = 416, Insurance = 229.

^1^Monthly household Income.

^2^In October 2017, 1 CHF = 0.97 US Dollar = 0.86 EUR.

**Fig. 1:**
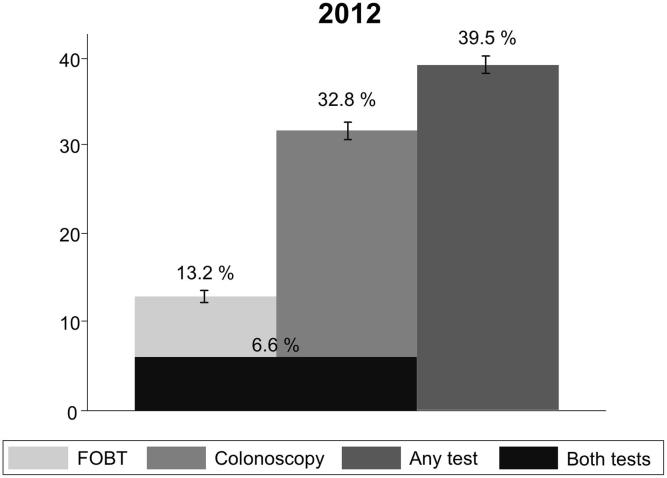
Weighted proportions of 50–75-year-old respondents tested for colorectal cancer in the Swiss Health Interview Survey 2012.

The weighted proportion of individuals tested for CRC within recommended intervals was 39.5% in 2012 (p < 0.001). 32.8% of individuals were tested with colonoscopy and 13.2% with FOBT (see Fig. 1). The unadjusted prevalence of FOBT and colonoscopy vary by nearly all covariates, except for nationality (see Supplemental File 2). Type of insurance and deductible were associated with colonoscopy but not with FOBT (type of insurance p = 0.79, deductible p = 0.53).

**Fig. 2:**
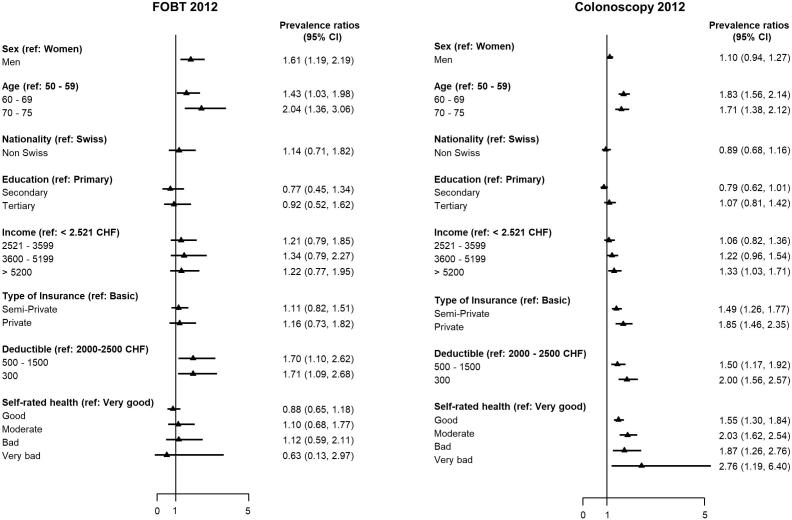
Adjusted prevalence ratios and 95% of confidence intervals (CI) of colorectal cancer testing by fecal occult blood testing (FOBT) within the past 2 years and colonoscopy in the past 10 years for the population of 50–75-year-olds, from the Swiss Health Interview Survey 2012.

Information on all covariates was available for 5869 respondents. Number of participants with missing values on covariates were 1199 for income, 1121 for deductible, 430 for insurance type, 103 for physician visits, 24 for education, 13 for self-rated status and 2 for education. These respondents were all included in our multivariate multinomial logistic regression models (see Supplemental File 3). Having the lowest deductible was significantly associated with Colonoscopy (PR 2.00, 95% CI: 1.56–2.57) and FOBT (PR 1.71, 95% CI: 1.09–2.68). Colonoscopy and private insurance were significantly associated (PR 1.85 95% CI:1.46 to 2.35); FOBT testing and private insurance were not (PR 1.12, 95% CI: 0.84–1.49). (see Supplemental File 3). Sex and age did not interact with type of insurance or deductible for either test (all P > 0.10) (see Fig. 2).

**Fig. 3:**
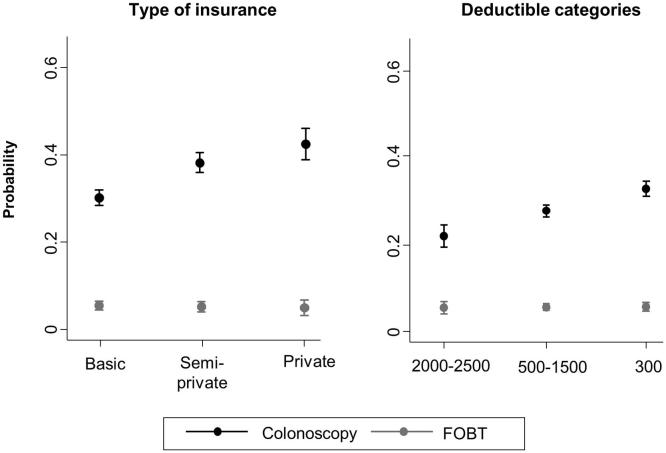
Predicted probabilities of CRC testing by FOBT within the last 2 years or colonoscopy within the last 10 years for a 60-year old man by type of insurance and deductible, from the Swiss Health Interview Survey 2012.

Fig. 3 illustrates the marginal proportion of having either test at each category type of insurance and level of deductible. A 60-year-old man with private insurance and the lowest deductible had a 28 percentage points greater probability of having been tested for CRC than a 60-year old man with basic insurance and the highest deductible (60% vs. 32%).

We used exploratory multinomial regression models that adjusted for the same covariates to classify respondents into screening and diagnostic FOBT and colonoscopy for the results of the 2012 Survey (Supplementary File 4). Type of insurance and deductible was associated with both screening and diagnostic colonoscopies. Screening FOBT was associated with deductible but not with private insurance. Diagnostic FOBT and was not associated with deductible or private insurance.

In total, 478/7342 (6.5%) reported changing their deductible in the last year. In sensitivity analyses, the point estimate of the measure of association between colonoscopy and semi-private insurance was 1.62 (95% CI: 1.31–1.91) when we excluded participants who changed their insurance status and 2.05 (95% CI: 1.58–2.68) when we compared the lowest to the highest deductible (Supplementary File 5).

When we restricted our analysis to colonoscopies performed within the year, the measure of association between colonoscopy and private insurance was 1.50 (95% CI: 1.06–2.11) and 1.76 (95% CI: 1.15–2.69) when we compared lowest to highest deductible (Supplementary File 6).

## 4 Discussion

Among the Swiss population, we found 40% of 50–75-year-olds had been tested for CRC. Colonoscopy, an expensive test, was significantly associated with private insurance and low deductible after multivariate adjustment for predictors of CRC testing (PR 1.88, 95% CI: 1.43–2.47). The cheaper FOBT was not significantly associated with insurance type but with deductible (PR 1.71, 95% CI: 1.09–2.68). In absolute terms, a 60-year-old Swiss man with private insurance and the lowest deductible had a 28 percentage points higher chance of having been tested for CRC than a 60-year-old man with basic insurance and the highest deductible (60% vs. 32%).

Among the Swiss population of 50- to 75-year-olds, we found 33% had been tested with colonoscopy within ten years, on the high end of the range for testing in countries outside the U.S., but lower than in Germany. A very early 2004/2005 Survey of Health, Aging and Retirement in Europe (SHARE) is still the largest survey of CRC testing in Europe (11 countries), and its findings are comparable to our findings in the SIHS (Stock and Brenner, 2010). SHARE found colonoscopy rates varied widely between countries, from 6.1% in Greece to 25.1% in France. A 2017 *meta*-analysis found colonoscopy use in countries ranged from 12% to 44% for lifetime colonoscopy, and 13–30% for recent colonoscopy (within 5–10 years), except for Germany where 55% of the screening-eligible population reported colonoscopies within the last 5–10 year, and the U.S., where 62% reported the same (Chen et al., 2017). A study conducted in Germany (Stock et al., 2011) analysed claims data from 2000 to 2008, and age-standardized their report of the percentage of individuals who had had colonoscopies within ≤10 years found 23% of men and 26% of women had been tested with colonoscopy.

We found 13% had been tested with FOBT within the last 2 years in Switzerland. SHARE found FOBT rates ranged from 4.1% in the Netherlands to 61.1% in Austria. The SHARE study included colonoscopy and FOBT tests in its questionnaire, but assessed FOBT within the last ten years. Our study assessed FOBT within recommended intervals (the last two years). Unfortunately, SHARE has since removed CRC screening questions, so we cannot track changes in rates over time, or directly compare rates in Switzerland to those in other countries after 2005. Chen et al.’s *meta*-analysis did not report on FOBT, but reported of the percentage of individuals who had FOBT within the last year and found 14% of men and 22% of women in Germany had been tested with FOBT (Stock et al., 2011). Our results affirm the increasing trend for colonoscopies and decreasing trend for FOBT Stock et al. also identified (Stock et al., 2011).

Our finding that testing rates and insurance type are associated agrees with the results of studies that suggest health insurance type is among the most important determinants of cancer screening. Data from the Women’s Health Initiative (WHI) showed that women who did not have prepaid health insurance plans were less likely to be screened for breast cancer (OR 0.30–0.67) (Hsia et al., 2000). Another U.S. study compared insured and uninsured patients and found that insured patients were more likely to be tested within guidelines for CRC testing (OR: 7.64) (Matthews et al., 2005). Basic insurance is mandatory in Switzerland, so 98% of the Swiss population is insured, but far fewer are eligible or opt for semi-private or private insurance or supplementary coverage (Biller-Andorno and Zeltner, 2015). The choice of more expensive semi-private and private policies and supplements may also be a proxy for additional financial resources.

Since testing rates and deductible were associated, we expect that Switzerland’s new reimbursement policy of 2013 will raise overall testing rates, and especially colonoscopy rates. When the 2012 SHIS was conducted, Switzerland reimbursed only diagnostic FOBT and colonoscopy prescribed for patients who showed CRC symptoms like abdominal pain, macroscopic blood in stool, or weight loss, or for patients with high CRC risk (personal or first-degree family history of CRC, or inflammatory bowel diseases). Patients who opted for screening with colonoscopy or FOBT may have had to pay out-of-pocket. Colonoscopy is at least ten times more expensive than FOBT, and this may have lowered the overall screening rate (Wharam et al., 2016). In July 2013, Switzerland began reimbursing colonoscopy screening every ten years or FOBT every two years for 50–69-year-olds, but we will not know if the new policy raised the number of people screened for CRC in the general population until the 2017 SIHS survey results become available in 2019. But even after the policy change, Swiss citizens must pay for CRC tests if they have not met their deductible, unless they participate in an organized, quality-assured program approved by the FOPH, in which deductibles have been waived for 50–69-year-olds since 2013. Various cantons in Switzerland will be launching such organised screening programs over the next years. By the end of the decade, we should be able to see if waiving the deductible lowers barriers for those with high deductible plans and mandatory basic insurance and if waiving the deductible raises overall CRC testing rates in Switzerland or changes the ratio of colonoscopy to FOBT tests.

Future studies should contain budget impact analyses (BIA) of the effects of organized screening programs on overall healthcare costs. Several cost-effectiveness studies contrasted the incremental cost-effectiveness ratio (ICER) of colonoscopy to that of FOBT, but came to different conclusions about the most cost-effective option over the long term (Lansdorp-Vogelaar et al., 2011). Their models were based on different assumptions about costs and benefits, varied in their time horizon, and in payer perspective. Most found that CRC screening saved between 2000 and 20,000 USD/life-year (range: cost-saving to 37,000 USD/life-years gained), falling into the commonly accepted range for cost-effective interventions (Lansdorp-Vogelaar et al., 2011). If our hypothesis that high deductible reduces the colonoscopy rate is true, eliminating the deductible for CRC screening will increase healthcare costs overall, but will be associated with better health outcomes. More important, eliminating the deductible for colonoscopy and FOBT would reduce the effect of out-of-pocket costs on the testing decisions of those invited for CRC screening, and increase the likelihood they will choose the screening option that best fits their preferences and values.

The Swiss healthcare system has some unique features that might affect CRC testing rates. Public and private hospital physicians, and self-employed specialists can schedule appointments in hospitals for semi-private or private patients and be paid extra for seeing them. Gastroenterologists are also paid extra for colonoscopies they perform in a hospital setting for these patients, which might provide incentive to perform more colonoscopies in this population.

### 4.1 Study limitations and strengths

Our study was strengthened by extensive data validation, control for non-response, and the SFSO’s complex weighting procedure, which ensured our sample represented the Swiss resident population. Adjusting for carefully selected, pre-specified determinants that reflect socio-economic, health care utilisation and health- and risk-related factors enabled us to highlight the association between insurance status and CRC testing. We were supported by good data collected via telephone interviews, but survey data may overestimate screening rates (Schneider et al., 2008). Though SHIS did not differentiate between sigmoidoscopy and colonoscopy (recommended screening intervals for the two methods differ), this may have had little effect because sigmoidoscopy is rare in Switzerland (Marbet et al., 2008). We also had to exclude some respondents from our adjusted model because they were missing co-variates, mainly for income or deductible, which might induce some selection bias. However, the proportion excluded was small, limiting the importance of this bias.

The SHIS asked participants their insurance status when they were surveyed, but did not ask what it was when they were tested. The data available did not allow us to assess the proportion of participants who changed deductible within the last 10 years. The SHIS did ask participants if they changed their deductible in the last year: in 2012, 6.5% did. This rate aligns with findings from a study in Switzerland based on administrative data from a health insurance. It estimated that, each year, 4.5% of the insured population changes their deductible (Gerfin et al., 2015). When we restricted our analyses to colonoscopies within the year (when the deductible participants who underwent colonoscopy could be expected to be same as it was when they were surveyed) the point estimate of the association between colonoscopy and insurance status was similar to that of the main analyses. Confidence intervals were wider because the number of reported colonoscopies was lower in the last 12 months than in the last 10 years.

In future, researchers should seek to analyze administrative data from health insurers, since insurers track deductibles on an individual level and over time.

We need to know if implementing organized screening programs in Switzerland reduced differences associated with insurance status, and differences in colonoscopy or FOBT rate by type of insurance, and we will test this after 2017 SHIS data becomes available. We also look forward to the release of data from another large survey, the European Health Interview Survey (EHIS), which included a question about colonoscopy in its 2013/2015 survey. Until this data is released we cannot determine if the trend we identified in Switzerland is consistent with trends in other European nations, or in Europe overall.

### 4.2 Conclusion

In 2012, about 60% of the Swiss population was not current with CRC testing. After adjusting for covariates, private insurance and low deductible were significantly associated with higher prevalence of CRC testing, indicating that waiving the deductible could increase CRC screening uptake and reduce health inequality.

## CRediT authorship contribution statement

**Leonhard Braun:** Conceptualization, Formal analysis, Writing - original draft. **Anja Kässner:** Formal analysis, Writing - review & editing. **Lamprini Syrogiannouli:** Formal analysis, Writing - review & editing. **Kevin Selby:** Methodology, Writing - review & editing. **Jean-Luc Bulliard:** Methodology, Writing - review & editing. **Yonas Martin:** Methodology, Writing - review & editing. **Idris Guessous:** Methodology, Writing - review & editing. **Kali Tal:** Methodology, Writing - review & editing. **Cinzia Del Giovane:** Formal analysis, Methodology, Writing - review & editing. **Marcel Zwahlen:** Formal analysis, Methodology, Writing - review & editing. **Reto Auer:** Conceptualization, Funding acquisition, Supervision, Project administration, Writing - review & editing.

## Declaration of Competing Interest

The authors declare that they have no known competing financial interests or personal relationships that could have appeared to influence the work reported in this paper.
