# Supplementary material for: Association between colorectal cancer testing and insurance type: Evidence from the Swiss Health Interview Survey 2012
Source: Prev Med Rep. 2020 May 4;19:101111. doi: 10.1016/j.pmedr.2020.101111 (PMC7226870; doi:10.1016/j.pmedr.2020.101111)
Supplement: Supplementary data 1 [file mmc1.docx]

**Supplementary File 1 – Directed acyclic graph of the association between non-modifiable factors and modifiable factors on CRC testing.**
