# Supplementary material for: Association between colorectal cancer testing and insurance type: Evidence from the Swiss Health Interview Survey 2012
Source: Prev Med Rep. 2020 May 4;19:101111. doi: 10.1016/j.pmedr.2020.101111 (PMC7226870; doi:10.1016/j.pmedr.2020.101111)
Supplement: Supplementary data 2 [file mmc2.docx]

**Supplementary File 2**- **Weighted unadjusted proportions of 50-75-year old respondents tested for colorectal cancer with fecal occult blood test (FOBT) only and colonoscopy (with or without FOBT) from the Swiss Health Interview Survey 2012.**

|  | Fecal occult blood testing in the past 2 years | | | Colonoscopy in the past 10 years | | |
| --- | --- | --- | --- | --- | --- | --- |
|  | % | 95% CI | p-value | % | 95% CI | p-value |

| Sex |  |  |  |  |  |  |
| --- | --- | --- | --- | --- | --- | --- |
| Male | 7.95 | 6.96 to 9.06 |  | 33.53 | 31.58 to 35.54 |  |
| Female | 5.43 | 4.42 to 6.65 | 0.002 | 32.06 | 30.24 to 33.95 | 0.288 |
| Age group |  |  |  |  |  |  |
| 50-59 | 6.01 | 4.85 to 7.42 |  | 25.99 | 24.05 to 28.03 |  |
| 60-69 | 6.81 | 5.82 to 7.95 |  | 38.32 | 36.14 to 40.55 |  |
| 70-75 | 8.12 | 6.63 to 10.09 | 0.122 | 38.81 | 35.62 to 42.10 | 0.000 |
| Nationality |  |  |  |  |  |  |
| Swiss | 6.48 | 5.80 to 7.23 |  | 33.35 | 31.97 to 34.77 |  |
| Non-Swiss | 7.69 | 5.21 to 11.21 | 0.400 | 29.77 | 25.76 to 34.13 | 0.121 |
| Education |  |  |  |  |  |  |
| Primary | 6.66 | 4.37 to 10.02 |  | 31.72 | 27.91 to 35.80 |  |
| Secondary | 6.31 | 5.48 to 7.26 |  | 31.73 | 30.04 to 33.46 |  |
| Tertiary | 7.26 | 6.08 to 8.65 | 0.623 | 35.30 | 32.79 to 37.90 | 0.091 |
| Income^1^ |  |  |  |  |  |  |
| <2521 CHF^2^ | 6.69 | 5.11 to 8.70 |  | 30.71 | 27.08 to 34.60 |  |
| 2521 – 3599 CHF | 6.99 | 5.49 to 8.87 |  | 29.47 | 26.48 to 32.65 |  |
| 3600 – 5199 CHF | 7.30 | 5.64 to 9.40 |  | 32.53 | 30.04 to 35.11 |  |
| >5200 CHF | 6.38 | 5.26 to 7.70 | 0.831 | 35.45 | 32.88 to 38.09 | 0.025 |
| Self-rated health |  |  |  |  |  |  |
| Very good | 7.40 | 6.17 to 8.85 |  | 25.74 | 23.56 to 28.04 |  |
| Good | 5.76 | 4.93 to 6.71 |  | 34.17 | 32.21 to 36.18 |  |
| Moderate | 7.37 | 5.20 to 10.34 |  | 39.50 | 36.03 to 43.08 |  |
| Bad | 9.50 | 6.31 to 14.06 |  | 37.10 | 30.61 to 44.09 |  |
| Very bad | 3.23 | 0.76 to 12.75 | 0.127 | 45.28 | 29.68 to 61.86 | 0.000 |
| Deductible |  |  |  |  |  |  |
| 2000 – 2500 CHF | 5.78 | 4.20 to 7.91 |  | 22.67 | 19.30 to 26.43 |  |
| 500 – 1500 CHF | 7.07 | 6.00 to 8.31 |  | 30.25 | 28.14 to 32.45 |  |
| 300 CHF | 7.01 | 5.83 to 8.42 | 0.534 | 38.29 | 36.22 to 40.41 | 0.000 |
| Type of insurance |  |  |  |  |  |  |
| Basic | 6.88 | 5.93 to 7.98 |  | 29.26 | 27.58 to 31.00 |  |
| Semi – private | 6.40 | 5.18 to 7.89 |  | 38.85 | 36.11 to 41.67 |  |
| Private | 6.32 | 4.59 to 8.65 | 0.789 | 43.58 | 39.51 to 47.74 | 0.000 |
| Note: missing values: nationality = 2, education = 24, income = 1,199, physician visits = 103, self-rated health status = 13, deductible = 1,121, insurance = 430, ^1^ monthly household Income, ^2^ In October 2017, 1 CHF = 0.97 US Dollar = 0.86 EUR | | | | | | |
