# Supplementary material for: Association between colorectal cancer testing and insurance type: Evidence from the Swiss Health Interview Survey 2012
Source: Prev Med Rep. 2020 May 4;19:101111. doi: 10.1016/j.pmedr.2020.101111 (PMC7226870; doi:10.1016/j.pmedr.2020.101111)
Supplement: Supplementary data 3 [file mmc3.docx]

**Supplementary File 3- Weighted adjusted prevalence ratios of colorectal cancer testing with fecal occult blood testing (FOBT) only and colonoscopy (with or without FOBT), from the Swiss Health Interview Survey 2012.**

|  | Fecal occult blood testing  in the past 2 years | | | Colonoscopy  in the past 10 years | | |
| --- | --- | --- | --- | --- | --- | --- |
|  | PR^1^ | 95%CI | p-value^2^ | PR^1^ | 95%CI | p-value^2^ |

| **Sex (ref: women)** |  |  |  |  |  |  |
| --- | --- | --- | --- | --- | --- | --- |
| Men | 1.61 | 1.19 to 2.19 | 0.002* | 1.10 | 0.94 to 1.27 | 0.225 |
| **Age (ref: 50-59)** |  |  |  |  |  |  |
| 60-69 | 1.43 | 1.03 to 1.98 |  | 1.83 | 1.56 to 2.14 |  |
| 70-75 | 2.04 | 1.36 to 3.06 | 0.003* | 1.71 | 1.38 to 2.12 | 0.000* |
| **Nationality (ref: Swiss)** |  |  |  |  |  |  |
| Not Swiss | 1.14 | 0.71 to 1.82 | 0.597 | 0.89 | 0.68 to 1.16 | 0.376 |
| **Monthly Income (ref: <2.521 CHF)** **^3, 4^** |  |  |  |  |  |  |
| 2521 - 3599 | 1.21 | 0.79 to 1.85 |  | 1.06 | 0.82 to 1.36 |  |
| 3600 - 5199 | 1.34 | 0.79 to 2.27 |  | 1.22 | 0.96 to 1.54 |  |
| >5200 | 1.22 | 0.77 to 1.95 | 0.743 | 1.33 | 1.03 to 1.71 | 0.082 |
| **Education (ref: Primary)** |  |  |  |  |  |  |
| Secondary | 0.77 | 0.45 to 1.34 |  | 0.79 | 0.62 to 1.01 |  |
| Tertiary | 0.92 | 0.52 to 1.62 | 0.403 | 1.07 | 0.81 to 1.42 | 0.001* |
| **Self-rated health (ref: very good)** |  |  |  |  |  |  |
| Good | 0.88 | 0.65 to 1.18 |  | 1.55 | 1.30 to 1.84 |  |
| Moderate | 1.10 | 0.68 to 1.77 |  | 2.03 | 1.62 to 2.54 |  |
| Bad | 1.12 | 0.59 to 2.11 |  | 1.87 | 1.26 to 2.76 |  |
| Very bad | 0.63 | 0.13 to 2.97 | 0.711 | 2.76 | 1.19 to 6.40 | 0.000* |
| **Type of Insurance (ref: Basic)** |  |  |  |  |  |  |
| Semi – private | 1.11 | 0.82 to 1.51 |  | 1.49 | 1.26 to 1.77 |  |
| Private | 1.16 | 0.73 to 1.82 | 0.719 | 1.85 | 1.46 to 2.35 | 0.000* |
| **Deductible (ref: 2000-2500 CHF)** **^4^** |  |  |  |  |  |  |
| 500 – 1500 | 1.70 | 1.10 to 2.62 |  | 1.50 | 1.17 to 1.92 |  |
| 300 | 1.71 | 1.09 to 2.68 | 0.043* | 2.00 | 1.56 to 2.57 | 0.000* |
| Note: Prevalence Ratios are adjusted for all variables in the table. N= 5869 ^1^ PR, Prevalence Ratios ^2^ We used the Wald test to generate p-value for the different groups ^3^ monthly household Income, ^4^ In October 2017, 1 CHF = 0.97 US Dollar = 0.86 EUR, ^5^ Visit in the last 12 Months*p-value <0.05 | | | | | | |
