# Supplementary material for: Association between colorectal cancer testing and insurance type: Evidence from the Swiss Health Interview Survey 2012
Source: Prev Med Rep. 2020 May 4;19:101111. doi: 10.1016/j.pmedr.2020.101111 (PMC7226870; doi:10.1016/j.pmedr.2020.101111)
Supplement: Supplementary data 4 [file mmc4.docx]

**Supplementary File 4 - adjusted and weighted prevalence ratios of colorectal cancer testing reported as screening or diagnostic for the population of 50-75-year-olds, from the Swiss Health Interview Survey 2012**

|  | **Tests reported as screening** | | | | | |  | **Tests reported as diagnostic** | | | | | |
| --- | --- | --- | --- | --- | --- | --- | --- | --- | --- | --- | --- | --- | --- |
|  | **FOBT** | | | **Colonoscopy** | | |  | **FOBT** | | | **Colonoscopy** | | |
|  | **PR^1^** | **95%CI** | **p-value^2^** | **PR^1^** | **95%CI** | **p-value^2^** |  | **PR^1^** | **95%CI** | **p-value^2^** | **PR^1^** | **95%CI** | **p-value^2^** |

| Sex (ref: Men) |  |  |  |  |  |  |  |  |  |  |  |  |  |
| --- | --- | --- | --- | --- | --- | --- | --- | --- | --- | --- | --- | --- | --- |
| Women | 0.59 | 0.46 to 0.76 | 0.000* | 0.64 | 0.52 to 0.77 | 0.000* |  | 1.66 | 0.87 to 3.20 | 0.127 | 1.34 | 1.12 to 1.61 | 0.001* |
| Age (ref: 50-59) |  |  |  |  |  |  |  |  |  |  |  |  |  |
| 60-69 | 1.47 | 1.11 to 1.94 |  | 2.00 | 1.63 to 2.47 |  |  | 0.89 | 0.46 to 1.73 |  | 1.30 | 1.07 to 1.58 |  |
| 70-75 | 2.29 | 1.64 to 3.19 | 0.000* | 1.96 | 1.49 to 2.58 | 0.000* |  | 0.70 | 0.25 to 1.94 | 0.787 | 1.19 | 0.93 to 1.53 | 0.027* |
| Nationality (ref: Swiss) |  |  |  |  |  |  |  |  |  |  |  |  |  |
| Not Swiss | 0.74 | 0.49 to 1.12 | 0.155 | 0.72 | 0.50 to 1.03 | 0.075 |  | 2.47 | 1.11 to 5.48 | 0.027* | 1.06 | 0.77 to 1.46 | 0.705 |
| Income (ref: <2.521 CHF) ^3, 4^ |  |  |  |  |  |  |  |  |  |  |  |  |  |
| 2521 - 3599 | 1.22 | 0.82 to 1.80 |  | 1.00 | 0.71 to 1.40 |  |  | 1.16 | 0.44 to 3.03 |  | 1.08 | 0.79 to 1.46 |  |
| 3600 - 5199 | 1.10 | 0.77 to 1.58 |  | 1.23 | 0.91 to 1.68 |  |  | 2.83 | 0.80 to 10.02 |  | 1.11 | 0.84 to 1.48 |  |
| >5200 | 1.12 | 0.77 to 1.63 | 0.804 | 1.43 | 1.03 to 1.97 | 0.041* |  | 2.06 | 0.68 to 6.24 | 0.345 | 1.09 | 0.81 to 1.49 | 0.904 |
| Education (ref: Primary) |  |  |  |  |  |  |  |  |  |  |  |  |  |
| Secondary | 0.96 | 0.65 to 1.44 |  | 0.71 | 0.52 to 0.98 |  |  | 0.47 | 0.18 to 1.21 |  | 0.95 | 0.71 to 1.28 |  |
| Tertiary | 1.19 | 0.77 to 1.83 | 0.322 | 0.82 | 0.58 to 1.16 | 0.079 |  | 0.41 | 0.15 to 1.11 | 0.201 | 1.31 | 0.93 to 1.84 | 0.011* |
| Self-rated health (ref: very good) |  |  |  |  |  |  |  |  |  |  |  |  |  |
| Good | 0.79 | 0.60 to 1.06 |  | 1.08 | 0.87 to 1.34 |  |  | 2.57 | 1.21 to 5.45 |  | 1.93 | 1.54 to 2.41 |  |
| Moderate | 0.74 | 0.52 to 1.07 |  | 0.88 | 0.65 to 1.19 |  |  | 5.30 | 2.04 to 13.77 |  | 3.03 | 2.32 to 3.95 |  |
| Bad | 0.80 | 0.45 to 1.43 |  | 0.93 | 0.57 to 1.53 |  |  | 8.12 | 2.76 to 23.91 |  | 2.78 | 1.81 to 4.27 |  |
| Very bad | 0.14 | 0.02 to 1.02 | 0.172 | 0.18 | 0.05 to 0.64 | 0.043* |  | 6.16 | 0.97 to 39.29 | 0.000* | 6.59 | 2.95 to 14.73 | 0.000* |
| Type of Insurance (ref: Basic) |  |  |  |  |  |  |  |  |  |  |  |  |  |
| Semi-Private | 1.28 | 0.96 to 1.70 |  | 1.63 | 1.32 to 2.02 |  |  | 0.76 | 0.41 to 1.42 |  | 1.18 | 0.96 to 1.40 |  |
| Private | 1.37 | 0.90 to 2..10 | 0.143 | 2.32 | 1.76 to 3.06 | 0.000* |  | 0.76 | 0.28 to 2.02 | 0.630 | 1.10 | 0.82 to 1.47 | 0.266 |
| Deductible (ref: 2000-2500 CHF) ^4^ |  |  |  |  |  |  |  |  |  |  |  |  |  |
| 500 – 1500 | 1.52 | 1.00 to 2.31 |  | 1.12 | 0.82 to 1.52 |  |  | 0.69 | 0.28 to 1.68 |  | 1.73 | 1.22 to 2.46 |  |
| 300 | 1.77 | 1.16 to 2.70 | 0.030* | 1.46 | 1.07 to 1.99 | 0.009* |  | 0.59 | 0.23 to 1.54 | 0.562 | 2.11 | 1.49 to 2.97 | 0.000* |
| ^1^ Prevalence ratios are adjusted for all variables in the table.^2^ We used the Wald test to generate p-values for the different groups. ^3^ monthly household Income, ^4^ In October 2017, 1 CHF = 0.97 US Dollar = 0.86 EUR, ^5^ in the last 12 Months, *p-value <0.05 | | | | | | | | | | | | | |
