# Supplementary material for: Association between colorectal cancer testing and insurance type: Evidence from the Swiss Health Interview Survey 2012
Source: Prev Med Rep. 2020 May 4;19:101111. doi: 10.1016/j.pmedr.2020.101111 (PMC7226870; doi:10.1016/j.pmedr.2020.101111)
Supplement: Supplementary data 5 [file mmc5.docx]

**Supplementary File 5 – Sensitivity analysis: Weighted adjusted prevalence ratios of colorectal cancer testing with fecal occult blood testing (FOBT) only and colonoscopy (with or without FOBT), excluding respondents who reported having changed their deductible in the last 12 months.**

|  | Fecal occult blood testing  in the past 2 years | | | Colonoscopy  in the past 10 years | | |
| --- | --- | --- | --- | --- | --- | --- |
|  | PR^1^ | 95%CI | p-value^2^ | PR^1^ | 95%CI | p-value^2^ |

| **Sex (ref: men)** |  |  |  |  |  |  |
| --- | --- | --- | --- | --- | --- | --- |
| Women | 0.59 | 0.43 to 0.81 | 0.001* | 0.90 | 0.77 to 1.05 | 0.178 |
| **Age (ref: 50-59)** |  |  |  |  |  |  |
| 60-69 | 1.42 | 1.02 to 1.99 |  | 1.78 | 1.51 to 2.10 |  |
| 70-75 | 2.00 | 1.32 to 3.03 | 0.005* | 1.68 | 1.35 to 2.10 | 0.000* |
| **Nationality (ref: Swiss)** |  |  |  |  |  |  |
| Not Swiss | 1.20 | 0.75 to 1.92 | 0.451 | 0.90 | 0.68 to 1.18 | 0.447 |
| **Monthly Income (ref: <2.521 CHF)** **^3, 4^** |  |  |  |  |  |  |
| 2521 - 3599 | 1.22 | 0.79 to 1.89 |  | 1.08 | 0.83 to 1.41 |  |
| 3600 - 5199 | 1.41 | 0.82 to 2.41 |  | 1.27 | 1.00 to 1.61 |  |
| >5200 | 1.28 | 0.80 to 2.08 | 0.660 | 1.40 | 1.08 to 1.81 | 0.035* |
| **Education (ref: Primary)** |  |  |  |  |  |  |
| Secondary | 0.75 | 0.43 to 1.29 |  | 0.81 | 0.63 to 1.04 |  |
| Tertiary | 0.85 | 0.48 to 1.50 | 0.478 | 1.07 | 0.80 to 1.43 | 0.004* |
| **Self-rated health (ref: very good)** |  |  |  |  |  |  |
| Good | 0.90 | 0.66 to 1.22 |  | 1.58 | 1.32 to 1.89 |  |
| Moderate | 1.11 | 0.68 to 1.82 |  | 2.06 | 1.63 to 2.61 |  |
| Bad | 1.19 | 0.63 to 2.24 |  | 1.99 | 1.34 to 2.96 |  |
| Very bad | 0.69 | 0.14 to 3.32 | 0.734 | 3.15 | 1.34 to 7.43 | 0.000* |
| **Type of Insurance (ref: Basic)** |  |  |  |  |  |  |
| Semi – private | 1.21 | 0.88 to 1.65 |  | 1.55 | 1.30 to 1.86 |  |
| private | 1.12 | 0.70 to 1.80 | 0.478 | 1.85 | 1.45 to 2.36 | 0.000* |
| **Deductible (ref: 2000-2500 CHF)** **^4^** |  |  |  |  |  |  |
| 500 – 1500 | 1.65 | 1.04 to 2.60 |  | 1.54 | 1.18 to 2.01 |  |
| 300 | 1.74 | 1.08 to 2.81 | 0.065 | 2.05 | 1.58 to 2.67 | 0.000* |
| Note: Prevalence Ratios are adjusted for all variables in the table. N= 5452 ^1^ PR, Prevalence Ratios ^2^ We used the Wald test to generate p-value for the different groups ^3^ monthly household Income, ^4^ In October 2017, 1 CHF = 0.97 US Dollar = 0.86 EUR, ^5^ Visit in the last 12 Months*p-value <0.05 | | | | | | |
