# Supplementary material for: Association between colorectal cancer testing and insurance type: Evidence from the Swiss Health Interview Survey 2012
Source: Prev Med Rep. 2020 May 4;19:101111. doi: 10.1016/j.pmedr.2020.101111 (PMC7226870; doi:10.1016/j.pmedr.2020.101111)
Supplement: Supplementary data 6 [file mmc6.docx]

**Supplementary File 6 – Sensitivity analysis: Weighted adjusted prevalence ratios of colorectal cancer testing with colonoscopy in the last year.**

|  | Colonoscopy in the last year | | |
| --- | --- | --- | --- |
|  | PR^1^ | 95%CI | p-value^2^ |

| **Sex (ref: men)** |  |  |  |
| --- | --- | --- | --- |
| Women | 0.86 | 0.68 to 1.09 | 0.207 |
| **Age (ref: 50-59)** |  |  |  |
| 60-69 | 1.51 | 1.18 to 1.94 |  |
| 70-75 | 1.13 | 0.81 to 1.57 | 0.003* |
| **Nationality (ref: Swiss)** |  |  |  |
| Not Swiss | 1.13 | 0.75 to 1.75 | 0.544 |
| **Monthly Income (ref: <2.521 CHF)** **^3, 4^** |  |  |  |
| 2521 - 3599 | 0.86 | 0.58 to 1.27 |  |
| 3600 - 5199 | 0.94 | 0.67 to 1.32 |  |
| >5200 | 1.16 | 0.80 to 1.68 | 0.349 |
| **Education (ref: Primary)** |  |  |  |
| Secondary | 0.91 | 0.63 to 1.30 |  |
| Tertiary | 0.81 | 0.54 to 1.23 | 0.589 |
| **Self-rated health (ref: very good)** |  |  |  |
| Good | 1.77 | 1.33 to 2.35 |  |
| Moderate | 1.94 | 1.36 to 2.76 |  |
| Bad | 3.08 | 1.85 to 5.14 |  |
| Very bad | 2.43 | 0.85 to 6.96 | 0.000* |
| **Type of Insurance (ref: Basic)** |  |  |  |
| Semi – private | 1.24 | 0.95 to 1.62 |  |
| Private | 1.50 | 1.06 to 2.11 | 0.005* |
| **Deductible (ref: 2000-2500 CHF)** **^4^** |  |  |  |
| 500 – 1500 | 1.44 | 0.94 to 2.20 |  |
| 300 | 1.76 | 1.15 to 2.69 | 0.022* |
| Note: Prevalence Ratios are adjusted for all variables in the table. N= 3805 ^1^ PR, Prevalence Ratios ^2^ We used the Wald test to generate p-value for the different groups ^3^ monthly household Income, ^4^ In October 2017, 1 CHF = 0.97 US Dollar = 0.86 EUR, ^5^ Visit in the last 12 Months*p-value <0.05 | | | |
